# Chemotherapy-induced cellular senescence suppresses progression of Notch-driven T-ALL

**DOI:** 10.1371/journal.pone.0224172

**Published:** 2019-10-29

**Authors:** Ying Zhang, Justin Gundelach, Lonnie D. Lindquist, Darren J. Baker, Jan van Deursen, Richard J. Bram

**Affiliations:** 1 Department of Pediatric and Adolescent Medicine, Mayo Clinic, Rochester, MN, United States of America; 2 Department of Pediatric and Adolescent Medicine, and Department of Biochemistry and Molecular Biology, Mayo Clinic, Rochester, MN, United States of America; 3 Department of Immunology, and Department of Pediatric and Adolescent Medicine, Mayo Clinic, Rochester, MN, United States of America; German Cancer Research Center (DKFZ), GERMANY

## Abstract

T cell acute lymphoblastic leukemia (T-ALL) is a serious hematologic malignancy that occurs in children and young adults. Current therapies include intensive chemotherapy and ionizing radiation that preferentially kill malignant cells. Unfortunately, they are frequently accompanied by unintended negative impacts, including the induction of cellular senescence and long-term toxicities in normal host tissues. Whether these senescent cells resulting from therapy increase the susceptibility to relapse or secondary cancers is unknown. Using transgenic and pharmacological approaches to eliminate doxorubicin-induced senescent cells in a Notch-driven T-ALL relapse mouse model, we find that these cells inhibit tumor recurrence, suggesting that senescence in response to treatment suppresses tumorigenesis. This finding, together with extensive evidence from others demonstrating that age-associated health problems develop dramatically earlier among childhood cancer survivors compared to age-matched counterparts, suggests a relationship between therapy-induced senescence and tumorigenesis. Although cancer risk is increased through accelerated premature-aging in the long run, therapy-induced senescence appears to protect survivors from recurrence, at least in the short run.

## Introduction

T-cell acute lymphoblastic leukemia (T-ALL) is a highly aggressive form of childhood cancer. Approximately 20% of patients with this disease fail to survive and many others suffer long-term side effects due to therapeutic intervention. The cell membrane receptor, Notch1, becomes constitutively activated by somatic mutation in 60% of cases of pediatric T-ALL[1]. Notch1 is composed of an extracellular subunit (NEC) and a transmembrane subunit (NTM). Through the binding of DSL family ligands, Notch1 is activated and subsequently cleaved by gamma-secretase. This cleavage releases the soluble Notch1 intracellular domain (ICN1), which translocates to the nucleus and alters transcription of target genes[2–4]. Transgenic mice that express ICN1 developed T cell lymphomas by 98 weeks. Leukemogenesis developed much faster, usually within 4 to 6 weeks, in mice that received bone marrow transduced with ICN1-expressing retrovirus[5].

Multiagent chemotherapy, and in some cases, cranial radiation, is currently used to treat pediatric T-ALL. Though the overall 5-year survival rate is approximately 80%, secondary cancer development or relapses of the original leukemia are two critical late adverse events that afflict some survivors of childhood cancer. Radiation and DNA-damaging chemotherapeutic agents induce senescence not only in normal cells, but also in certain cancer cells[6–11]. Senescent cells stably exit the cell cycle, which is considered an important physiological barrier in suppressing tumor initiation and progression. Additionally, senescent cells are often characterized by increased secretion of different cytokines, inflammatory factors, growth factors and other proteins, together described as the Senescence Associated Secretory Phenotype (SASP)[12]. The SASP can promote senescence in neighboring cells, which may further enhance tumor suppression. Paradoxically, senescence has also been proposed to have pro-tumorigenic effects via secretion of other cytokines and growth factors that may promote the proliferation of tumor cells, in a context-dependent fashion[13].

In clinical trials, chemotherapy induced senescence impacts both tumor cells and host defenses simultaneously, making it challenging to dissect out the cell autonomous from non-autonomous effects. To specifically explore whether senescence induced by potent chemotherapy impairs host factors that would block relapse of T-ALL, we pre-treated mice prior to receiving a miniscule inoculum of leukemia cells. We used genetic and pharmacological methods of eliminating senescent cells to examine the role of doxorubicin-induced senescent cells in the development of Notch-driven T-ALL.

## Materials and methods

### Animal models

p16+/Luc mice, generated by the Sharpless lab[14], have a luciferase reporter gene knocked-in to the endogenous p16-INK4A locus. p16 INK-ATTAC mice that carry the *ink-ATTAC* transgene have been previously reported[15]. This strain expresses the green fluorescent protein (EGFP) and an inducible suicide gene (FK506-binding-protein–caspase 8, also known as ATTAC for “apoptosis through targeted activation of caspase”) under control of a fragment of the p16-INK4A promoter that is activated in senescent cells. Both GFP and ATTAC proteins are expressed selectively in senescent cells in these transgenic mice. C57BL/6 mice were purchased from the Jackson Laboratory (000664). Mice were housed under pathogen-free conditions. All animal work were approved by the Mayo Clinic Institutional Animal Care and Use Committee (IACUC Protocol A16715-15 titled as “Uncovering pathogenesis and novel treatment strategies for leukemia” and IACUC Protocol A00002817-17 titled as “Role of Cellular Senescence in Late Effects of Childhood Cancer Therapy”).

### Retroviral vectors and bone marrow transductions

MigR1-ICN1-GFP retrovirus that expresses activated NOTCH ICN1 and GFP from an IRES was previously reported[16]. The expression is under the control of MSCV promoter. PLAT-E cells were used to package the retroviral vectors for transductions were a kind gift from Dr. Kay Medina (Department of Immunology, Mayo Clinic). Cells were cultured in high glucose DMEM under antibiotic selection with 1 μg/ml puromycin and 10 μg/ml blasticidin (Sigma-Aldrich). PLAT-E cells were transfected with 12 μg retroviral vector MigR1-ICN1 using Lipofectamine 2000 (Life Technologies). Viral supernatants were collected 48–72 h post transfection and filtered (0.45 μm) prior to use.

### Relapse model of T-ALL

Bone marrow stem cells harvested from 5-FU–treated mice were transduced with retroviral vector MigR1-ICN1. We first employed Dr. Warren Pear’s method[17] of generating T-ALL in mice by transplanting the transduced bone marrow into lethally irradiated (800 rads) wild-type C57BL/6 recipient mice. We then isolated the resulting leukemic cells from spleens of moribund mice, and preserved them by freezing in aliquots in DMSO-containing medium. Defined numbers of stored T-ALL cells were intravenously injected into cohorts of mice. All the mice developed systemic T-ALL over a period of 4–6 weeks. The mice that received the lowest number (5) of T-ALL cells were selected as a relapse model for the subsequent experiments.

### Senescent cell induction and clearance

Mice were treated with 2 doses of doxorubicin (10 μg/g body weight, Selleckchem.com) or vehicle only. The gap between 2 doses was either 2 weeks or 3 weeks. During the 3 weeks resting period after doxorubicin treatment, p16 INK-ATTAC mice received the ATTAC-activating dimer AP20187 (2.5 μg/g body weight, Clontec) or vehicle by IP (intraperitoneal) injection. C57BL/6 mice were fed with 100 mg/kg ABT263 in vehicle (50% honey in PBS with 15% DMSO/7% Tween-20) through voluntary ingestion[18]. For feeding, single-use syringes (1 mL) with a ball-tipped gastric-feeding needle (Codan Medical, Rodby, Denmark) were used.

### Flow cytometry

Mouse peripheral blood was collected from the tail and facial vein. Erythrocytes were removed by brief treatment of ACK (Ammonium-Chloride-Potassium) Lysing Buffer. GFP signals of white blood cells or adipose cells in p16 INK-ATTAC mice were analyzed using an Accuri C6 flow cytometer (BD Biosciences, San Jose, CA, USA). Cell cycle was measured by C6 flow cytometer (FL2-A) after fixing cells with 70% ethanol and staining with propidium iodide (PI). Red blood cells in the spleen were eliminated by Lympholyte®-M (Cedarlane, CL5035). GFP signals and CD4, CD8 surface staining of splenocytes in p16 +/Luc mice infected with MigR1-ICN1-GFP were monitored by C6 flow cytometer as well. Antibodies were from BD Biosciences—Pharmingen. Cells were stained for surface expression of CD4 and CD8. Live cell counting was conducted by collecting equal volumes of cells and gated based on forward and side scatter profiles.

### Senescence-associated-β-galactosidase (SA-β-Gal) staining:

SA-β-Gal staining of mouse inguinal adipose tissue was performed using a kit according to the manufacturers’ instructions (Cell Signaling). Whole mouse inguinal adipose tissue were excised and stored in PBS on ice until fixation. Adipose tissue were fixed for 15 min at room temperature, washed twice in PBS, and developed in staining solution for 24 h at 37°C.

### qRT-PCR

Total RNA was extracted from lymphocytes collected from peripheral blood using a Qiagen RNeasy plus mini kit, (Cat#: 74134) according to the manufacturer’s protocol. Transcription into cDNA was performed using Oligo-dT and SuperScript III reverse transcriptase (Invitrogen, Cat#: 18080–400) according to the manufacturer’s instructions. All PCR reactions used SYBR green PCR Master Mix (Applied Biosystems) to a final volume of 12 μl, with each cDNA sample performed in triplicate in the ABI PRISM7900 Sequence Detection System (Applied Biosystems) according to the protocol of the manufacturer. The expression of genes was normalized to 18S rRNA. Sequences of primers for 18S rRNA, p21, IL6, and Mmp13 were as follows: 18S rRNA forward: 5’-CGCTTCCTTACCTGGTTGAT-3’, 18S rRNA reverse: 5’-GAGCGACCAAAGGAACCATA-3’; p21 forward: 5’-GTCCAATCCTGGTGATGTCC-3’, p21 reverse: 5’-GTTTTCGGCCCTGAGATGT-3’; IL6 forward: 5’-GACAACTTTGGCATTGTGG-3’, IL6 reverse: 5’-ATGCAGGGATGATGTTCTG-3’; Mmp13 forward: 5’-CTGGACCAAACTATGGTGGG-3’,; Mmp13 reverse: 5’-GGTCCTTGGAGTGATCCAGA-3’.

### Statistical analysis

Prism software was used for the generation of all survival curves and statistical analyses.

## Results

### Cellular senescence is induced by doxorubicin in p16 INK-ATTAC mice

p16 INK-ATTAC transgenic mice express FKBP-Casp8 (also known as ATTAC) fusion protein and GFP under control of the minimal p16-INK4A promoter[15]. This allele, commonly referred as Ink4a/Arf or Cdkn2a, is a biomarker of cellular senescence. In the BubR1 progeroid (hypomorphic, BubR1^H/H^) mouse background, inguinal adipose tissue (IAT) of untreated BubR1^H/H^; INK-ATTAC mice stained strongly for SA-β-Gal. GFP positive cells isolated from the IAT expressed significantly higher levels of p16Ink4a and other key senescence markers including p21, p19, Pai1, IL6, and Igfbp2[15] than those of wild-type controls. These properties indicated that GFP expression can be reliably used to detect and isolate p16 Ink4a–positive senescent cells from p16 INK-ATTAC mice.

**Fig 1:**
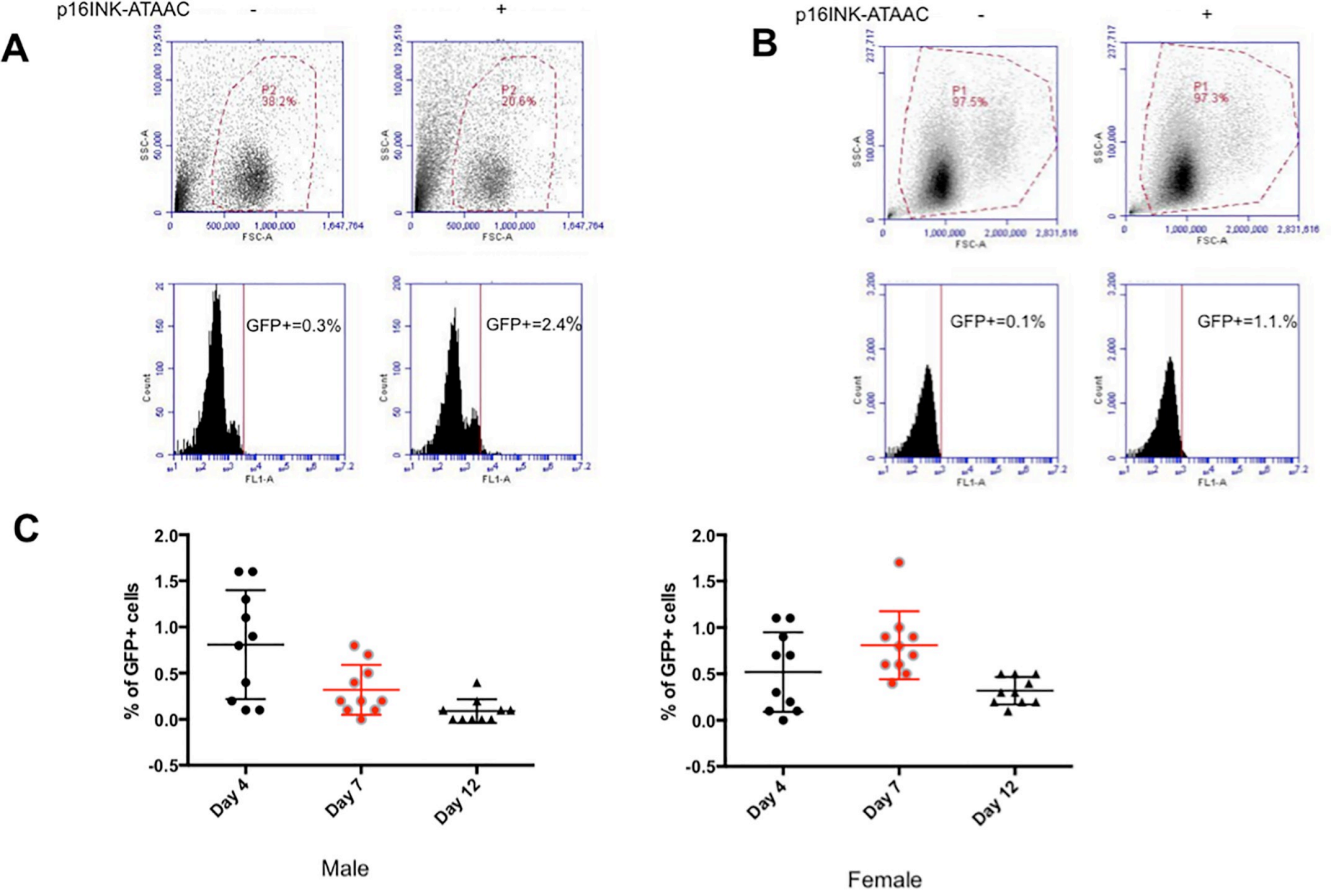
p16 INK-ATTAC+ mouse is an established mouse model studying cellular senescence. (A) GFP positive cells were detected in adipose tissue of p16 INK-ATTAC+ mouse [FL1: 530±15nm (FITC/GFP)]. (B) Senescent cells were observed in white blood cells after two doses of doxorubicin treatment in p16 INK-ATTAC mice. (C) The dynamics of the senescent cells population post-chemo (Female: n = 10; male: n = 10).

First, we harvested IAT from 8-month-old p16 INK-ATTAC mice and looked for GFP positive cells by flow cytometry. As shown previously[15], we observed a distinct GFP positive cell population in single cell suspensions indicating the presence of senescent cells from fat (Fig 1A). To determine if chemotherapy induces senescence in p16 INK-ATTAC mice, we treated them with the anthracycline drug doxorubicin twice, separated by a 2-week interval. We observed increased numbers of GFP positive cells in peripheral blood samples compared to those from control wild type mice. (Fig 1B), confirming the induction of cellular senescence. The ability to track p16-expressing senescent cells in the peripheral blood gave us the opportunity to monitor the dynamics of these populations over time. Therefore, we measured GFP positive cells in 10 male and 10 female mice at days 4, 7 and 12 after a single dose of doxorubicin. We found that GFP positive cell populations peaked at day 4 in male and at day 7 in female mice (Fig 1B). Taken together, these results indicated that cellular senescence could be observed in p16 INK-ATTAC mouse white blood cells following doxorubicin treatment.

### Establishment of a NOTCH-induced T-ALL relapse model by intravenous injection of leukemic cells

**Fig 2:**
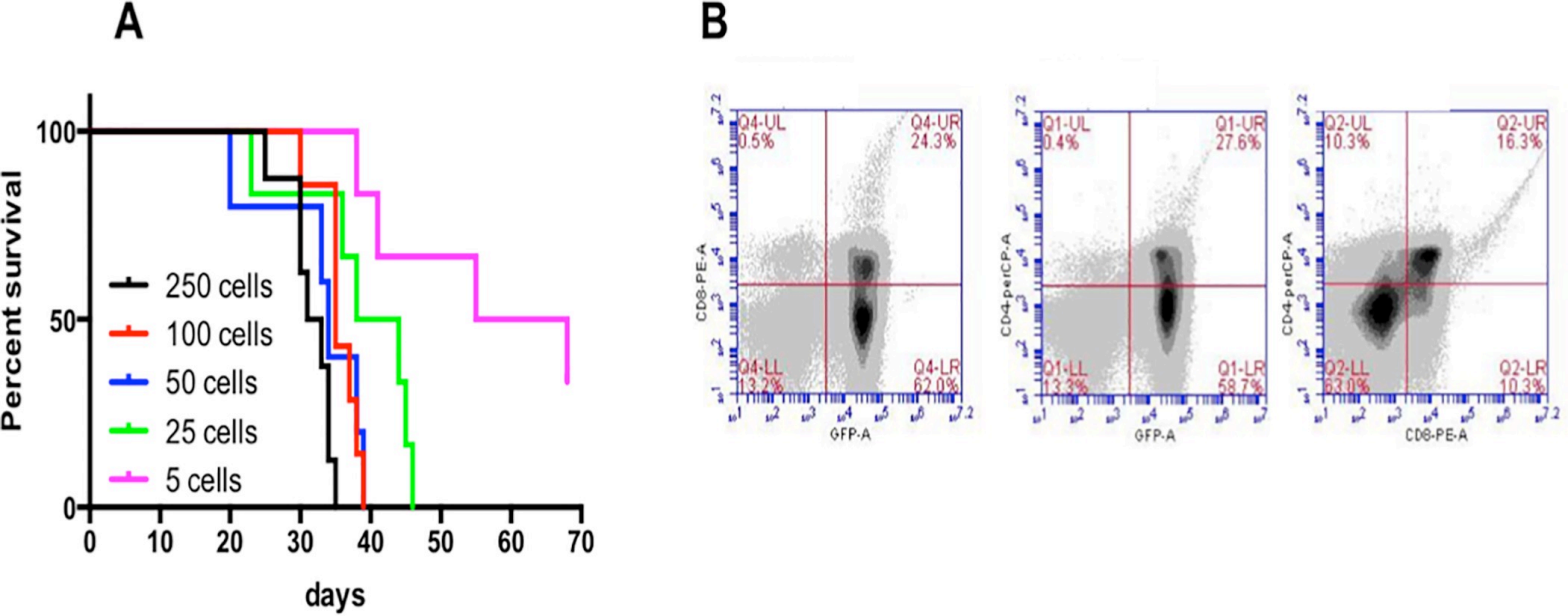
Leukemogenesis was generated by direct injection of splenic ICN-1-induced T-ALL cells. (A) Kaplan-Meier survival curve of p16+/Luc mice receiving 250, 100, 50, 25, and 5 T-ALL cells. 6 mice were for each dose of injections, including 3 females and 3 males. (B) GFP analysis and CD4+CD8+ surface staining of splenocytes. Numbers in each dot plot refer to the percentages in each quadrant. Cells that express GFP from the Notch1-expressing retrovirus represent T-ALL cells.

To specifically examine the ability of wild type mice to resist recurrence of leukemia, we developed a novel relapse model that started with very low numbers of tumor cells. Moribund mice previously transplanted with MigR1-ICN1 expressing bone marrows were euthanized, and their leukemic cells were isolated from their engorged spleens. Cells were preserved in DMSO-containing medium and stored in aliquots at -150°C. After thawing, we intravenously injected a broad range of T-ALL cells (250, 100, 50, 25, or 5 cells) into cohorts of p16 +/Luc, Albino C57BL/6 recipient mice. Impressively, all mice, even those receiving as few as 5 cells, developed systemic T-ALL over a period of 4–6 weeks (Fig 2A), with the median survival of recipient mice being related to the number of injected cells. As mentioned earlier, T-ALL cells express GFP from the Notch1-expressing retrovirus, therefore, we used the GFP signal in blood cells to monitor the development of T-ALL. The analysis of GFP expression and CD4/CD8 surface markers of the splenocytes collected from ill-appearing mice (Fig 2B) verified that Notch-induced T-ALL could be adoptively transferred through intravenous injection of a minute amount of leukemic cells in the absence of leukodepletion in the recipients. Mice that received the least number (5) had the longest median survival (55 days). Since the low number of injected cell recapitulates the onset of tumor recurrence, we used those mice as a relapse model for the subsequent experiments.

### Clearance of chemo-induced senescent cells accelerates the development of T-ALL in p16 ink-ATACC mice

Previous studies[15, 19] indicated that, in naturally aging mice and in BubR1 progeroid mice, p16 Ink4a-positive senescent cells can be efficiently cleared by administration of AP20187 (AP), a small molecule dimerizer that activates the FKBP-fused Caspase 8 to initiate apoptosis. Induced clearance of senescence cells delayed aging-associated phenotypes, including the incidence of lordokyphosis and cataracts, in these mouse models. At the molecular level, AP-treated mice demonstrated a significant loss of SA-β -Gal positive IAT cells and decreased senescence-associated markers compared with the IAT of untreated counterparts.

**Fig 3:**
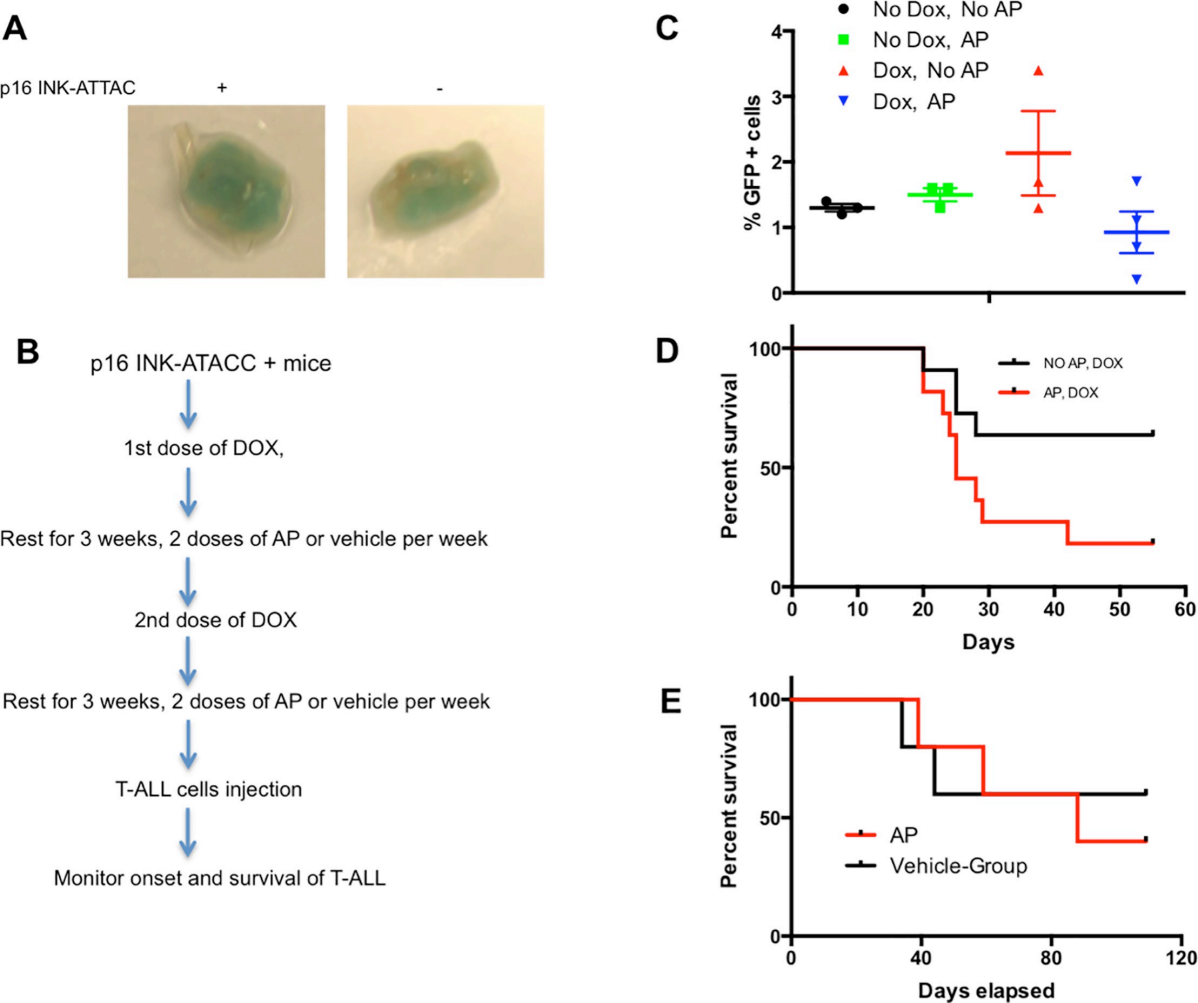
Clearance of chemo-induced senescent cell accelerated the development of T-ALL in p16 INK-ATACC mice. (A) SA-β -Gal stained IAT collected from 10- month-old p16 INK-ATTAC+ (left) and p16 INK-ATTAC- (right) mice. Both mice received AP20187. (B) Schematic of experimental procedures for (C) and (D). (C) Senescent (GFP+) cells were observed in peripheral blood on day 5 after the 2^nd^ dose of doxorubicin (10 mg/kg) followed by the treatment plan indicated in Fig 3B. Each group had 3 female mice. (D) Kaplan-Meier survival curve of p16 INK-ATTAC+ mice receiving doxorubicin and AP (2.5 mg/kg). The 1^st^ cohort (Black line, NO AP, DOX) received doxorubicin only (n = 11); the 2^nd^ cohort (Red line, AP, DOX) received both doxorubicin and AP20187 (n = 11). Log-rank (Mantel-Cox) test, *p<0*.*05*. (E) AP itself does not have an impact on the development of T-ALL. AP group: n = 5; Vehicle group: n = 5.

To confirm the ability of AP to eliminate senescent cells in our model, we performed SA-β -Gal staining on IAT collected from AP-treated-10- month-old p16 INK-ATTAC+ (Fig 3A, left) and p16 INK-ATTAC- mice (Fig 3A, right). A marked decrease in SA-β -Gal was seen in p16 INK-ATTAC+ mice compared with the IAT of AP-treated wild type mice. There was also a clear trend that AP reduced the population of GFP-positive cells induced by doxorubicin (Fig 3C). These results, together with previous findings[15, 19], led us to design a protocol to allow testing of the impact of therapy-induced senescence on susceptibility to T-ALL relapse (Fig 3B). Two cohorts of 3 to 6 month old p16 INK-ATTAC+ mice, including males and females, were used. The 1^st^ cohort (NO AP, DOX) received two cycles of doxorubicin separated by a 3-week rest, and followed by another 3-week rest period. The 2^rd^ cohort (AP, DOX) received the same two cycles of doxorubicin, followed by twice weekly injections of AP during both 3-week rest periods, in order to clear the accumulation of senescent cells induced by doxorubicin. After this treatment, mice were injected with a very low number of Notch-induced T-ALL cells to mimic a state of minimal residual disease. The mice were then closely monitored for the onset and the progression of leukemic disease. Mice that became moribund were euthanized and the time interval between innoculation of cells to the time of euthanasia of the animals was recorded. Kaplan-Meier survival curve analysis (Fig 3D) indicated that mice with reduced burden of senescent cells (AP-treated cohort) developed T cell leukemia faster than the cohort of mice that received doxorubicin but no AP-dimer (p<0.05 by Mantel-Cox log-rank analysis). As a control to rule out the possibility that AP alone promoted the development of T-ALL, we also treated mice with AP or vehicle only after injecting T-ALL cells. No differences were observed in their survival (Fig 3E).

### ABT-263-mediated senescent cell clearance promotes T-ALL progression

It has been shown that cellular senescence, at least in part, contributes to the development of age-related diseases[20]. Moreover, by removing senescence cells, physical function at older ages could be improved along with extended lifespan[15, 19, 21, 22]. These findings have led to efforts to develop “senolytic” drugs, compounds that preferentially induce apoptosis of senescent cells in order to suppress aging-associated diseases[23]. ABT263 (also known as navitoclax), a specific inhibitor of the anti-apoptotic proteins BCL-2 and BCL-xL[24], was identified as a potent senolytic drug that selectively kills senescent cells in culture and in mice. The clearance of senescent cells by ABT263 not only dramatically rejuvenates aged hematopoietic stem cells in mice but also reduces the burden, number, and sizes of plaques in an atherosclerotic mouse model[6, 25].

**Fig 4:**
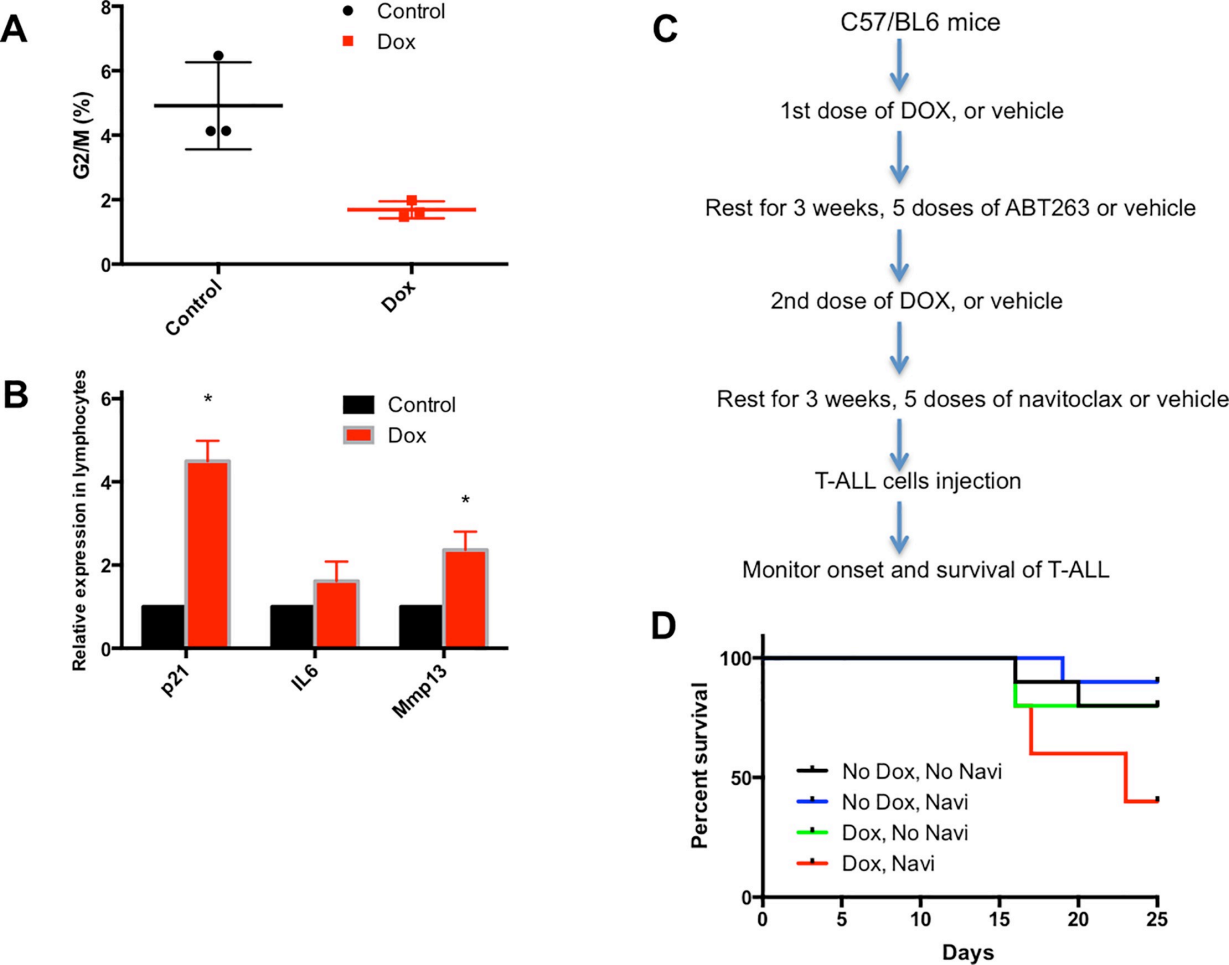
Elimination of senescent cells by senolytic drug (ABT263, also known as Navitoclax) promotes T-ALL progression. (A) Percentages of G2/M phase cells in the peripheral blood of control or doxorubicin-treated C57/BL6 mice (Control: n = 3; Dox: n = 3. *p<0*.*05 by two-tailed unpaired t-test)*. (B) Relative expression of senescent marker mRNAs specific for *p21*, *IL6*, and *Mmp13* in blood cells from control or doxorubicin-treated mice. Control: n = 3; Dox: n = 4. *p<0.*01*. (C) Schematic of experimental procedures for (D). (D) Kaplan-Meier survival curve of C57/BL6 mice receiving doxorubicin and navitoclax (50 mg/kg). The 1^st^ cohort (Black line, No Dox, No Navi) only received vehicle (n = 10); the 2^nd^ cohort (Blue line, No Dox, Navi) received navitoclax only (n = 10); the 3^rd^ cohort (Green line, Dox, No Navi) received doxorubicin only (n = 10). The 4^th^ cohort (Red line, Dox, Navi) received both doxorubicin and navitoclax (n = 10). Logrank test for trend, *p<0*.*05*.

Before we tested this distinct mechanism to kill senescent cells, we analyzed the cell cycle of the peripheral blood lymphocyte subpopulation stained with propidium iodide in C57BL/6 mice. Although most cells were in G1, we did observe a significant further reduction in the populations of G2/M cells after doxorubicin treatment (Fig 4A). We then compared the gene expression of several senescent markers, including *p21*, *IL6*, and *Mmp13* in the absence or presence of doxorubicin through qRT-PCR. As indicated in Fig 4B, p21 and Mmp13 levels were significantly higher compared to those of vehicle-only control mice, further consistent with the idea that doxorubicin induces senescence in this non-transgenic mouse model as well.

To further evaluate the impact of ablating cellular senescence by senolytic drugs, we repeated the T-ALL relapse assay using C57BL/6 mice treated with either vehicle or ABT263 during the two 3-week resting periods following doxorubicin treatment (Fig 4C). In line with our findings in AP-treated p16 INK-ATTAC+ mice, depletion of senescent cells in doxorubicin-treated mice by ABT263 accelerated the progression of Notch-induced T-ALL compared to controls (green and red lines, Fig 4D). On the other hand, the survival curves of mice treated with either vehicle or ABT263 lacking doxorubicin-treatment were not different (black and blue lines, Fig 4D). We conclude that ABT263 on its own does not accelerate the growth of subsequently injected T-ALL cells, and that the anti-tumor effect mediated by cellular senescence after chemotherapy treatment can have a significant impact in this relapse model.

## Discussion

In this study, we generated a novel T-ALL mouse model that mimics a part of the early stages of tumor relapse by injecting mice with very low number of leukemia cells and monitoring time to development of full blown leukemia. With the assistance of this model, we have studied the role of therapy-induced senescent cells in tumorigenesis. In order to avoid the possible interference with neoplasia-induced senescence, mice in this model system were not primed with T-ALL prior to treatment with chemotherapeutic drugs. This allowed us to study in isolation the innate (non-adaptive) processes that resist progression of leukemia from a very early stage.

Using both transgenic and pharmacological approaches to clear doxorubicin-induced senescent cells, we demonstrated that clearance of senescent cells accelerated the recurrence of Notch-induced-T-ALL, suggesting that senescence generated during treatment may indeed play an important role in the suppression of tumor development. Although the precise mechanism by which doxorubicin-induced senescence could have anti-tumorigenic activities is not yet clear, we suspect it could result from arresting the proliferation of damaged or dysfunctional cells or recruiting certain types of innate immune cells to constrain the malignant progression of tumor. This work lends support to the notion that senescence is an important physiological barrier in inhibiting tumor initiation and progression.

Being an unexpected part of the therapeutic effect, senescent cells appear to accumulate in aging pediatric cancer survivors. As a possible consequence, higher prevalence and earlier onset of multiple aging-associated health problems are commonly seen among childhood cancer survivors than in age-matched individuals in the general population. Although cancer can occur at any age, advancing age is one of the most important risk factors for cancer overall, and for many different cancer types. Therefore premature aging resulting from aggressive treatment might be one of the major factors that drive the onset of second malignancies among long-term childhood cancer survivors in later years after their cancer diagnosis.

**Fig 5:**
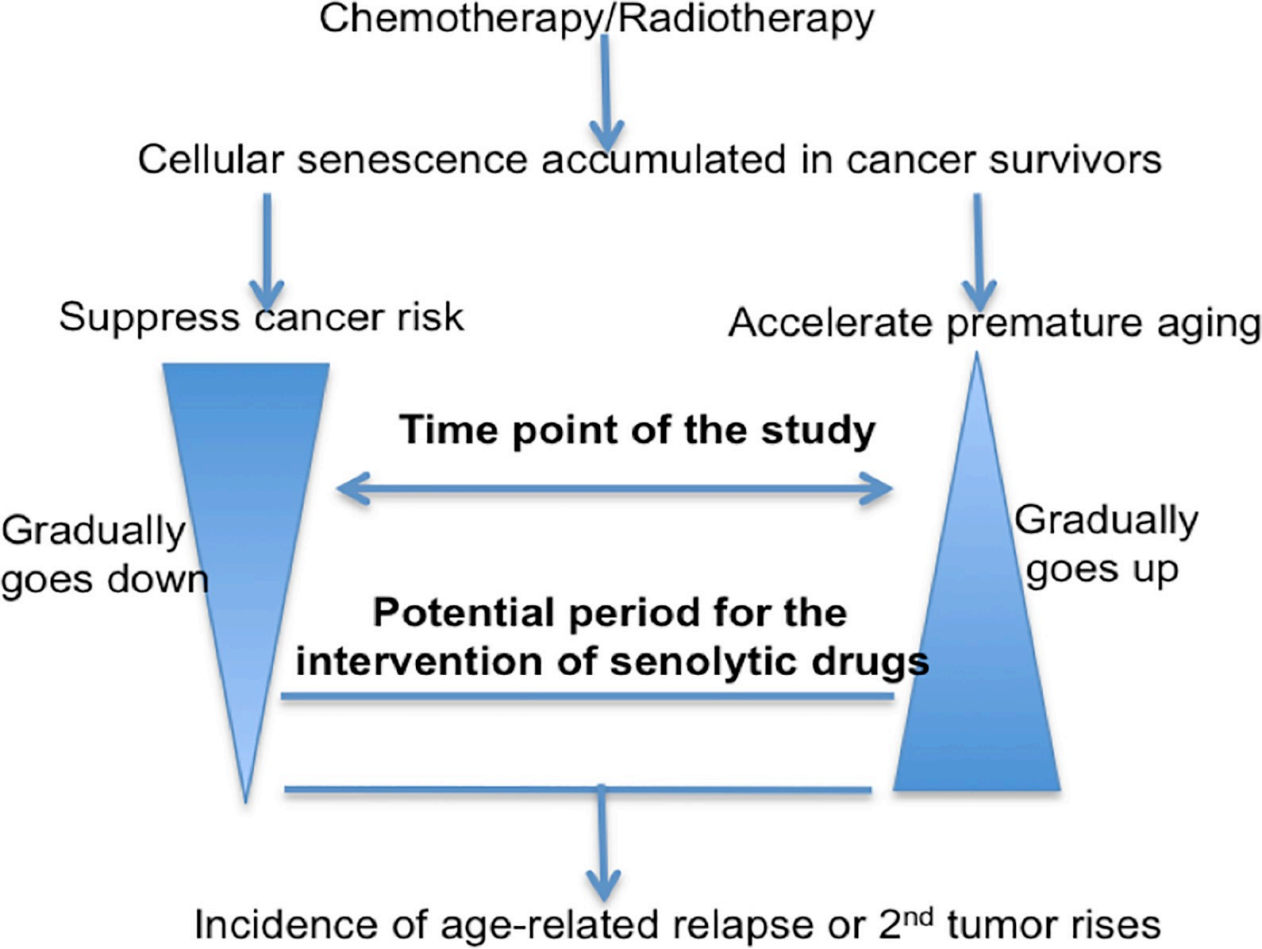
Model for the role of therapy-induced senescence in tumorigenesis. Senescence accumulated after chemotherapy/Radiotherapy leads to suppression of cancer and premature aging. As therapy-recipients age, the impact on the suppression gradually goes down, whereas the impact on aging gradually goes up. After reaching certain point, age-related cancer incidence rises in the long-term cancer survivors. Time point of our study and potential period for the intervention of senolytic drugs in order to reduce cancer incidence are indicated in the graph.

To align our conclusions that doxorubicin-induced senescent cells inhibit the development of T-ALL with evidence for significantly accelerated aging observed in cancer survivors as shown by others, we suggest a model for the role of therapy-induced senescence in tumorigenesis (Fig 5). Senescence resulting from cancer-therapy may thus be a double-edged sword, capable of suppressing cancer as well accelerating premature aging. We suspect that this inhibitory effect may be potent initially but then gradually disappears, as cancer survivors become older. Simultaneously, the induction of pre-mature aging may increase over time. The time frame of our studies was focused at a point where senescent cells are more active in tumor suppression. However, once the impact of aging on potential risks for cancer start to become dominant, senescent cells may instead develop pro-tumor effects, leading to elevated malignancy rates in long-term cancer survivors. Although further studies and more clinical work will be required to test this model, it raises the intriguing possibility that the intervention of senolytic drugs starting at certain time points could be beneficial for the long-term pediatric cancer survivors.

Our study indicates that the enhancement of pro-senescence therapy is likely able to reduce the risk of relapse in the short run, however, in the long run, anti-cancer effects due to cellular senescence will likely be overcome by increased rates of aging. It is important therefore that monitoring cellular senescence and choosing appropriate times to start the administration of senolytic drugs will be crucial for long-term survivors in order to minimize detrimental effects. It is estimated that over 100,000 children in the U.S. are cancer survivors, with the expected addition of approximately 12,000 young patients annually. Unfortunately, how therapy-induced senescent cells influence cancer survivors remains an open question. Answers to this question will help these survivors minimize long-term toxicities and improve their quality of life.
